# Supplementary material for: Evaluation of Urinary miRNA in Renal Cell Carcinoma: A Systematic Review
Source: Cancers (Basel). 2025 Apr 16;17(8):1336. doi: 10.3390/cancers17081336 (PMC12026426; doi:10.3390/cancers17081336)
Supplement: Supplementary file 1 [file cancers-17-01336-s001.zip › cancers-3516229-supplementary.pdf]

Supplementary Table 1: PRISMA (Preferred Reporting Items for Systematic Reviews and Meta-Analyses) checklist 2020.

Section and Topic	Item #	Checklist item	Location where item is reported (pages)
**TITLE**			
Title	1	Identify the report as a systematic review.	1
**ABSTRACT**			
Abstract	2	See the PRISMA 2020 for Abstracts checklist.	1-2
**INTRODUCTION**			
Rationale	3	Describe the rationale for the review in the context of existing knowledge.	2
Objectives	4	Provide an explicit statement of the objective(s) or question(s) the review addresses.	3
**METHODS**			
Eligibility criteria	5	Specify the inclusion and exclusion criteria for the review and how studies were grouped for the syntheses.	3
Information sources	6	Specify all databases, registers, websites, organisations, reference lists and other sources searched or consulted to identify studies. Specify the date when each source was last searched or consulted.	4
Search strategy	7	Present the full search strategies for all databases, registers and websites, including any filters and limits used.	4
Selection process	8	Specify the methods used to decide whether a study met the inclusion criteria of the review, including how many reviewers screened each record and each report retrieved, whether they worked independently, and if applicable, details of automation tools used in the process.	4
Data collection process	9	Specify the methods used to collect data from reports, including how many reviewers collected data from each report, whether they worked independently, any processes for obtaining or confirming data from study investigators, and if applicable, details of automation tools used in the process.	4
Data items	10a	List and define all outcomes for which data were sought. Specify whether all results that were compatible with each outcome domain in each study were sought (e.g. for all measures, time points, analyses), and if not, the methods used to decide which results to collect.	4
	10b	List and define all other variables for which data were sought (e.g. participant and intervention characteristics, funding sources). Describe any assumptions made about any missing or unclear information.	-
Study risk of bias assessment	11	Specify the methods used to assess risk of bias in the included studies, including details of the tool(s) used, how many reviewers assessed each study and whether they worked independently, and if applicable, details of automation tools used in the process.	4
Effect measures	12	Specify for each outcome the effect measure(s) (e.g. risk ratio, mean difference) used in the synthesis or presentation of results.	4
Synthesis methods	13a	Describe the processes used to decide which studies were eligible for each synthesis (e.g. tabulating the study intervention characteristics and comparing against the planned groups for each synthesis (item #5)).	-
	13b	Describe any methods required to prepare the data for presentation or synthesis, such as handling of missing summary statistics, or data conversions.	-
	13c	Describe any methods used to tabulate or visually display results of individual studies and syntheses.	-
	13d	Describe any methods used to synthesize results and provide a rationale for the choice(s). If meta-analysis was performed, describe the model(s), method(s) to identify the presence and extent of statistical heterogeneity, and software package(s) used.	4

Section and Topic	Item #	Checklist item	Location where item is reported (pages)
	13e	Describe any methods used to explore possible causes of heterogeneity among study results (e.g. subgroup analysis, meta-regression).	4
	13f	Describe any sensitivity analyses conducted to assess robustness of the synthesized results.	-
Reporting bias assessment	14	Describe any methods used to assess risk of bias due to missing results in a synthesis (arising from reporting biases).	4
Certainty assessment	15	Describe any methods used to assess certainty (or confidence) in the body of evidence for an outcome.	4-5
**RESULTS**			
Study selection	16a	Describe the results of the search and selection process, from the number of records identified in the search to the number of studies included in the review, ideally using a flow diagram.	5, 7
	16b	Cite studies that might appear to meet the inclusion criteria, but which were excluded, and explain why they were excluded.	5
Study characteristics	17	Cite each included study and present its characteristics.	5-6, 8-9
Risk of bias in studies	18	Present assessments of risk of bias for each included study.	6, 10
Results of individual studies	19	For all outcomes, present, for each study: (a) summary statistics for each group (where appropriate) and (b) an effect estimate and its precision (e.g. confidence/credible interval), ideally using structured tables or plots.	8-9
Results of syntheses	20a	For each synthesis, briefly summarise the characteristics and risk of bias among contributing studies.	8-9
	20b	Present results of all statistical syntheses conducted. If meta-analysis was done, present for each the summary estimate and its precision (e.g. confidence/credible interval) and measures of statistical heterogeneity. If comparing groups, describe the direction of the effect.	8-9
	20c	Present results of all investigations of possible causes of heterogeneity among study results.	8-9
	20d	Present results of all sensitivity analyses conducted to assess the robustness of the synthesized results.	-
Reporting biases	21	Present assessments of risk of bias due to missing results (arising from reporting biases) for each synthesis assessed.	10
Certainty of evidence	22	Present assessments of certainty (or confidence) in the body of evidence for each outcome assessed.	6, 10
**DISCUSSION**			
Discussion	23a	Provide a general interpretation of the results in the context of other evidence.	11
	23b	Discuss any limitations of the evidence included in the review.	11-12
	23c	Discuss any limitations of the review processes used.	12
	23d	Discuss implications of the results for practice, policy, and future research.	12
**OTHER INFORMATION**			
Registration and protocol	24a	Provide registration information for the review, including register name and registration number, or state that the review was not registered.	3
	24b	Indicate where the review protocol can be accessed, or state that a protocol was not prepared.	3

Section and Topic	Item #	Checklist item	Location where item is reported (pages)
	24c	Describe and explain any amendments to information provided at registration or in the protocol.	-
Support	25	Describe sources of financial or non-financial support for the review, and the role of the funders or sponsors in the review.	15
Competing interests	26	Declare any competing interests of review authors.	-
Availability of data, code and other materials	27	Report which of the following are publicly available and where they can be found: template data collection forms; data extracted from included studies; data used for all analyses; analytic code; any other materials used in the review.	-

2

3 Supplementary Table 2: SWiM (Synthesis without metanalysis) checklist.

**SWiM is intended to complement and be used as an extension to PRISMA**			
**SWiM reporting item**	**Item description**	**Page in manuscript where item is reported**	**Other**
Methods			
**1** Grouping studies for synthesis	1a) Provide a description of, and rationale for, the groups used in the synthesis (e.g., groupings of populations, interventions, outcomes, study design)	-	-
	1b) Detail and provide rationale for any changes made subsequent to the protocol in the groups used in the synthesis	-	-
**2** Describe the standardised metric and transformation methods used	Describe the standardised metric for each outcome. Explain why the metric(s) was chosen, and describe any methods used to transform the intervention effects, as reported in the study, to the standardised metric, citing any methodological guidance consulted	4	-
**3** Describe the synthesis methods	Describe and justify the methods used to synthesise the effects for each outcome when it was not possible to undertake a meta-analysis of effect estimates	4	-
**4** Criteria used to prioritise results for summary and synthesis	Where applicable, provide the criteria used, with supporting justification, to select the particular studies, or a particular study, for the main synthesis or to draw conclusions from the synthesis (e.g., based on study design, risk of bias assessments, directness in relation to the review question)	4	5

SWiM reporting item	Item description	Page in manuscript where item is reported	-
**5** Investigation of heterogeneity in reported effects	State the method(s) used to examine heterogeneity in reported effects when it was not possible to undertake a meta-analysis of effect estimates and its extensions to investigate heterogeneity	4	-
**6** Certainty of evidence	Describe the methods used to assess certainty of the synthesis findings	4	5
**7** Data presentation methods	Describe the graphical and tabular methods used to present the effects (e.g., tables, forest plots, harvest plots).<br>Specify key study characteristics (e.g., study design, risk of bias) used to order the studies, in the text and any tables or graphs, clearly referencing the studies included	-	-
Results			
**8** Reporting results	For each comparison and outcome, provide a description of the synthesised findings, and the certainty of the findings. Describe the result in language that is consistent with the question the synthesis addresses, and indicate which studies contribute to the synthesis	5-10	-
Discussion			
**9** Limitations of the synthesis	Report the limitations of the synthesis methods used and/or the groupings used in the synthesis, and how these affect the conclusions that can be drawn in relation to the original review question	11-12	-

4

Supplementary Table 3: List of excluded full text.

Title	Authors	Publication year	Reason for exclusion
Circle-Seq reveals genomic and disease-specific hallmarks in urinary cell-free extrachromosomal circular DNAs.	Lv, Wei;Pan, Xiaoguang;Han, Peng;Wang, Ziyu;Feng, Weijia;Xing, Xue;Wang, Qingqing;Qu, Kunli;Zeng, Yuchen;Zhang, Cailin;Xu, Zhe;Li, Yi;Zheng, Tianyu;Lin, Ling;Liu, Chengxun;Liu, Xuemei;Li, Hanbo;Henriksen, Rasmus Amund;Bolund, Lars;Lin, Lin;Jin, Xin;Yang, Huanming;Zhang, Xiuqing;Yin, Tailang;Regenberg, Birgitte;He, Fan;Luo, Yonglun	2022	Wrong outcome
ELF5 drives angiogenesis suppression though stabilizing WDTC1 in renal cell carcinoma.	Li, Tushuai;Xu, Longjiang;Wei, Zhe;Zhang, Shaomei;Liu, Xingyu;Yang, Yanzi;Gu, Yue;Zhang, Jie	2023	Wrong outcome
MicroRNA-204-5p: A novel candidate urinary biomarker of Xp11.2 translocation renal cell carcinoma.	Kurahashi, Ryoma;Kadomatsu, Tsuyoshi;Baba, Masaya;Hara, Chiaki;Itoh, Hitoshi;Miyata, Keishi;Endo, Motoyoshi;Morinaga, Jun;Terada, Kazutoyo;Araki, Kimi;Eto, Masatoshi;Schmidt, Laura S;Kamba, Tomomi;Linehan, W Marston;Oike, Yuichi	2019	Wrong population
MicroRNAs in Body Fluids: A More Promising Biomarker for Clear Cell Renal Cell Carcinoma.	Shi, Lei;Wang, Mengheng;Li, Haiping;You, Pengtao	2021	Wrong study design
LncRNA RCAT1 promotes tumor progression and metastasis via miR-214-5p/E2F2 axis in renal cell carcinoma.	Guo, Renbo;Zou, Benkui;Liang, Yiran;Bian, Jiasheng;Xu, Jian;Zhou, Qian;Zhang, Chao;Chen, Tao;Yang, Mingshan;Wang, Huansheng;Pei, Fajun;Xu, Zhonghua	2021	Wrong outcome
Urinary-derived extracellular vesicle microRNAs as non-invasive diagnostic biomarkers for early-stage renal cell carcinoma.	Zhang, Yu;Zhu, Yuan-Yuan;Chen, Yang;Zhang, Lele;Wang, Rong;Ding, Xiaoyu;Zhang, Huizi;Zhang, Chen-Yu;Zhang, Chunni;Gu, Wan-Jian;Wang, Cheng;Wang, Jun-Jun	2024	Wrong outcome
New Progress of Epigenetic Biomarkers in Urological Cancer.	Wu, Peng;Cao, Ziyi;Wu, Song	2016	Wrong outcome
miR-224-5p Contained in Urinary Extracellular Vesicles Regulates PD-L1 Expression by Inhibiting Cyclin D1 in Renal Cell Carcinoma Cells.	Qin, Zhiyuan;Hu, Haihong;Sun, Wen;Chen, Lu;Jin, Shengnan;Xu, Qingwen;Liu, Yuxi;Yu, Lushan;Zeng, Su	2021	Wrong outcome
Prognostic urinary miRNAs for the assessment of small renal masses.	Di Meo, Ashley;Brown, Marshall D;Finelli, Antonio;Jewett, Michael A S;Diamandis, Eleftherios P;Yousef, George M	2020	Wrong comparator
An increased level of MiR-222-3p is associated with TMP2 suppression, ERK activation and is associated with metastasis and a poor prognosis in renal clear cell carcinoma.	Lyu, Jia;Zhu, Yongzhe;Zhang, Qi	2020	Wrong outcome
Urinary exosome miR-30c-5p as a biomarker of clear cell renal cell carcinoma that inhibits progression by targeting HSPA5.	Song, Shangqing;Long, Manmei;Yu, Guopeng;Cheng, Yajun;Yang, Qing;Liu, Jiayi;Wang, Yiwei;Sheng, Jiayan;Wang, Linhui;Wang, Zhong;Xu, Bin	2019	Wrong outcome

High efficiency capture of biomarker miRNA15a for noninvasive diagnosis of malignant kidney tumors.	Renner, Alexander M;Derichsweiler, Christina;Ilyas, Shaista;Gessner, Isabel;Fries, Jochen W U;Mathur, Sanjay	2022	Wrong outcome
Exosomal MicroRNAs Are Diagnostic Biomarkers and Can Mediate Cell-Cell Communication in Renal Cell Carcinoma.	Butz, Henriett;Nofech-Mozes, Roy;Ding, Qiang;Khella, Heba W Z;Szabó, Peter M;Jewett, Michael;Finelli, Antonio;Lee, Jason;Ordon, Michael;Stewart, Robert;Krylov, Sergey;Yousef, George M	2016	Wrong outcome
Systematic Investigation of Immune-Related lncRNA Landscape Reveals a Potential Long Non-Coding RNA Signature for Predicting Prognosis in Renal Cell Carcinoma.	Liu, Kepu;Li, Zhibin;Ruan, Dongli;Wang, Huilong;Wang, Wei;Zhang, Geng	2022	Wrong outcome
MiRNA extraction from cell-free biofluid using protein corona formed around carboxyl magnetic nanoparticles.	Xu, Shengqiang;Nasr, Seyedmehdi Hossaini;Chen, Daoyang;Zhang, Xiaoxian;Sun, Liangliang;Huang, Xuefei;Qian, Chunqi	2018	Wrong study design
Urinary miRNAs Predict Metastasis in Patients With Clinically Localized Clear Cell Renal Cell Carcinoma Treated With Nephrectomy.	Borges Dos Reis, Rodolfo;Shu, Xiang;Ye, Yuanqing;Borregales, Leonardo;Karam, Jose A;Adibi, Mehad;Wu, Xifeng;Reis, Leonardo O;Wood, Christopher G	2024	Wrong comparator
Smartphone-Based Fluorescent Profiling of Quaternary MicroRNAs in Urine for Rapid Diagnosis of Urological Cancers Using a Multiplexed Isothermal Exponential Amplification Reaction.	Yao, Chanyu;Liu, Xueliang;Lu, Xiaohui;Wang, Lei;Jia, Jia;Li, Zheng	2024	Wrong study design
MicroRNA 15a, inversely correlated to PKCα, is a potential marker to differentiate between benign and malignant renal tumors in biopsy and urine samples.	von Brandenstein, Melanie;Pandarakalam, Jency J;Kroon, Lukas;Loeser, Heike;Herden, Jan;Braun, Gabriele;Wendland, Katherina;Dienes, Hans P;Engelmann, Udo;Fries, Jochen W U	2012	Wrong study design
